# Can oblique lateral interbody fusion (OLIF) create more lumbosacral lordosis in lumbar spine surgery than minimally invasive transforaminal interbody fusion (MIS-TLIF)?

**DOI:** 10.3389/fsurg.2022.1063354

**Published:** 2023-01-06

**Authors:** Jie Li, Yilei Chen, Hao Wu, Kaifeng Gan, Dikai Bei, Tengdi Fan, Jian Chen, Fengdong Zhao, Binhui Chen

**Affiliations:** ^1^ Department of Orthopaedic Surgery, Ningbo Medical Center Li Huili Hospital, Ningbo, Zhejiang, China; ^2^ Department of Orthopaedic Surgery, Sir Run Run Shaw Hospital, Zhejiang University School of Medicine; Key Laboratory of Musculoskeletal System Degeneration and Regeneration Translational Research of Zhejiang Province, Hangzhou, China; ^3^ Department of Orthopaedics and Traumatology, The University of Hong Kong, Hong Kong, China

**Keywords:** OLIF, MIS-TLIF, posterior pedicle screw fixation, OLIF standalone, lumbar degenerative disease, lumbosacral lordosis

## Abstract

**Objective:**

To compare the differences in the correction effect for lumbosacral lordosis and clinical outcomes between OLIF with/without posterior pedicle screw fixation (PSF) and MIS-TLIF through a retrospective cohort study.

**Method:**

There were 98 consecutive patients originally enrolled for the study, but 15 patients were excluded due to intraoperative endplate injury or osteotomy performed for severe spinal deformity. Thus, 83 patients included in this study (36 males and 47 females, mean age 66.0 ± 10.8 years) underwent single to three-segment OLIF (including OLIF + PSF and OLIF Standalone) or MIS-TLIF surgery from 2016 to 2018. The operation time, bleeding and blood transfusion, fusion rate, complication, pre-and postoperative visual analogue scale (VAS), Oswestry Disability Index (ODI) were evaluated. In addition, radiological parameters including lumbosacral lordosis (LL), fused segment lordosis (FSL), anterior disc height (ADH) and posterior disc height (PDH) were measured. The clinical outcomes, LL, FSL, ADH and PDH restored and were compared between the OLIF group, OLIF subgroups and MIS-TLIF group.

**Results:**

The average operation time and intraoperative bleeding were significantly less in the OLIF group than in the MIS-TLIF group (189 ± 83 vs. 229 ± 80 min, 113 ± 138 vs. 421 ± 210 ml), *P *< 0.001). There was no statistically significant difference between the OLIF group and the MIS-TLIF group in VAS and ODI improvements, fusion rate, complication, LL and FSL correction (*P *> 0.05). The ADH and PDH increases in the OLIF group were more than that in MIS-TLIF group (*P *< 0.001). The correction of LL was significantly more in the OLIF+PSF group than in the MIS-TLIF group (10.6 ± 8.7 vs. 4.0 ± 6.1 deg, *P* = 0.005).

**Conclusion:**

OLIF and MIS-TLIF are both safe and effective procedures, capable of restoring lumbosacral lordosis and disc height partly. Combined with PSF, OLIF can achieve a better correction effect of lumbosacral lordosis than MIS-TLIF.

## Level of evidence

Level III.

## Introduction

As a lumbar interbody fusion (LIF) approach, minimally invasive transforaminal interbody fusion (MIS-TLIF) has long been widely used to treat a variety of degenerative lumbar diseases (1). Although an effective procedure, MIS-TLIF still interferes with the posterior paravertebral muscles and spinal canal. In recent years, with the rapid development of anterior spinal instrumentation, anterior/lateral interbody fusion has been increasingly used as an alternative to conventional posterior surgery (2, 3). The anterior approach allows direct access to the disc from the anterior (ALIF) or lateral (LLIF) side and the implantation of a larger cage to better restore the physiological lordosis of the lumbar spine. Thus, ALIF and LLIF have a theoretical advantage over the posterior approach in restoring spinal sagittal alignment and disc height (4, 5).

LLIF is currently the mainstream anterior fusion technique at the lumbar segment except for L5/S1, which can be divided into standard lateral approach (XLIF/DLIF) and oblique lateral approach (OLIF) according to the channel direction. OLIF has a lower risk of lumbar plexus and psoas muscle injury compared with X/DLIF (6). However, at present, the comparison of the correction effect for lumbosacral lordosis between OLIF combined with PSF, OLIF Standalone and MIS-TLIF in lumbar degenerative diseases is rarely reported. Therefore, we aimed to assess the correction effect for lumbosacral lordosis and clinical outcomes of OLIF with/without adding posterior PSF (OLIF Standalone) and MIS-TLIF and evaluate the effect of additional PSF on the lordosis correction of OLIF. We hypothesize that OLIF with additional posterior pedicle screw fixation (PSF) provides a better correction effect for lumbosacral lordosis and comparable clinical outcomes compared with MIS-TLIF.

## Material and methods

### Study design

Ethical approval was obtained from the Medical Ethics Committee of the hospital.

**Figure 1:**
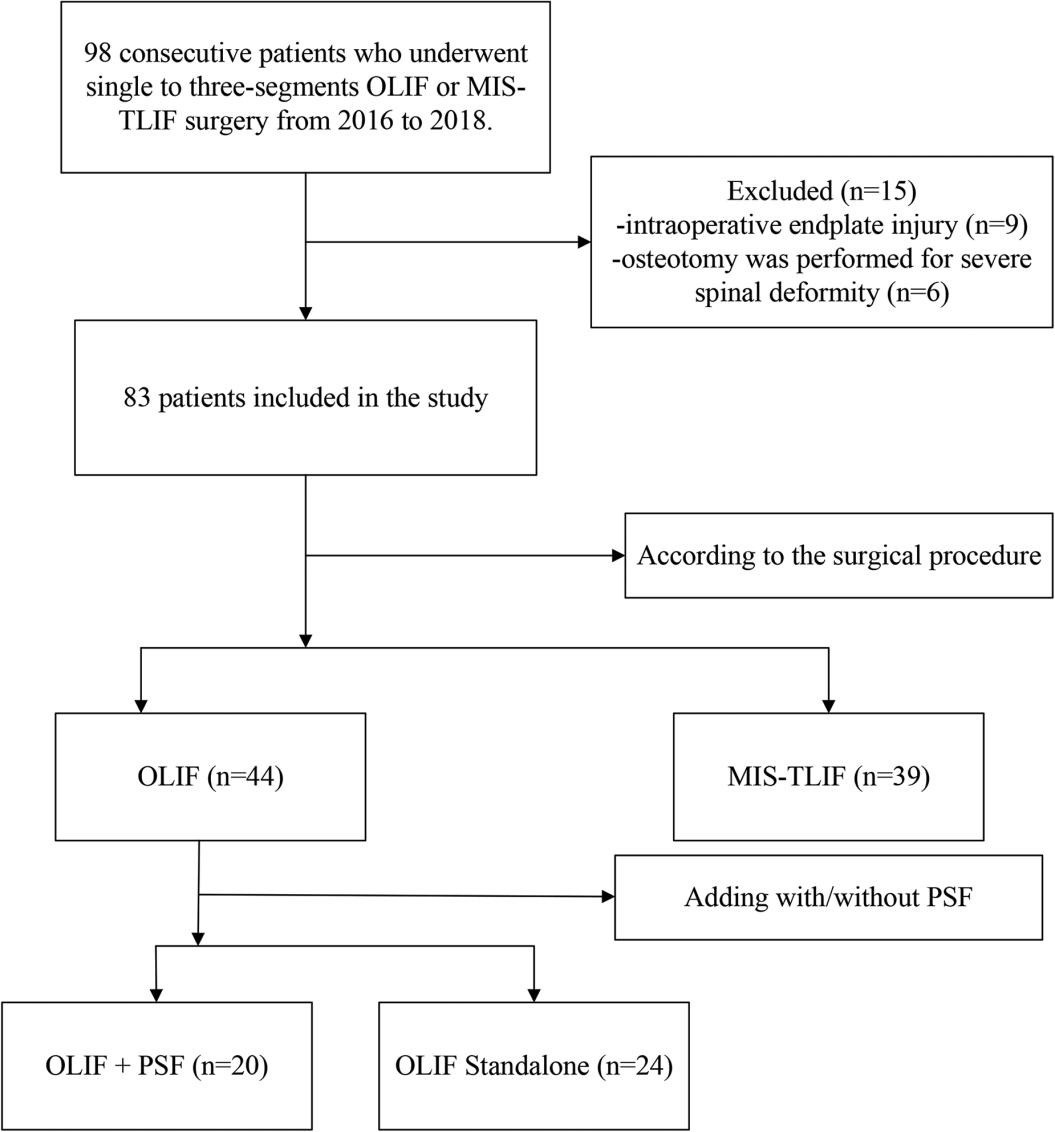
A flowchart of patients included in the study.

Additionally, all patients gave written informed consent for their information to be stored in the hospital's database and used for the study. Inclusion criteria: (1) Cases with the lumbar degenerative disease treated with OLIF or TLIF who had failed conservative treatment. (2) The number of fused segments was one to three. Exclusion criteria: (1) Revision surgery. (2) Previous lumbar fusion at other segments. (3) Intraoperative endplate injury and cage collapse. (4) Severe scoliosis or sagittal imbalance that required an osteotomy. There were 98 consecutive patients originally enrolled for the study, but 15 patients were excluded due to intraoperative endplate injury (*n* = 9) or osteotomy performed for the severe spinal deformity (*n* = 6). Consequently, 83 consecutive patients included in this study (36 males and 47 females, mean age 65.8 years) underwent single to three-segment OLIF (including OLIF + PSF and OLIF Standalone) or MIS-TLIF surgery from 2016 to 2018 (Figure 1). There were 9 cases of lumbar disc herniation with calcification, 39 cases of lumbar spinal stenosis, 22 cases of degenerative lumbar spondylolisthesis, 12 cases of degenerative lumbar scoliosis, and 1 case of lumbar isthmic spondylolisthesis. According to the surgical procedure, the OLIF group (44 cases including 20 cases of OLIF + PSF, 24 cases of OLIF Standalone) and TLIF group (39 cases) were divided into two groups. There were 33 cases of single-segment fusion, 27 cases of double-segment fusion, and 23 cases of triple-segment fusion. Posterior fixation was performed in 59 cases, and no posterior fixation was performed in 24 cases.

### Surgical techniques

**Figure 2:**
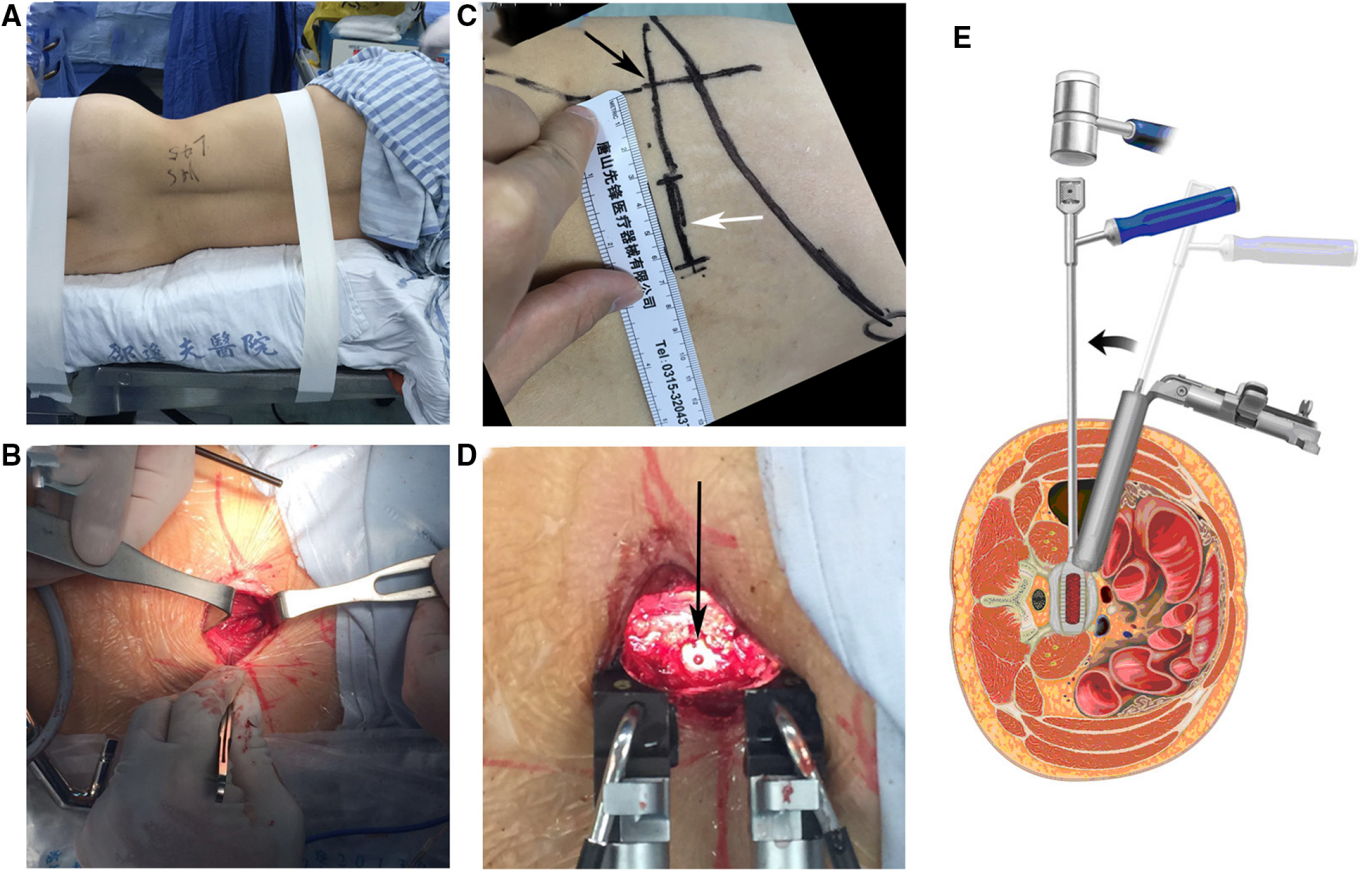
OLIF procedure (**A**) OLIF position: right side prone position. (**B**) OLIF incision: preoperative fluoroscopic localization of the center of the target disc (black arrow), about 3-4 cm anterior to the midpoint of the target disc and 4 cm in length (white arrow). (**C**) Sequential separation of the abdominal muscles in the direction of the muscle fibers. (**D**) After completion of cage (arrow) implantation. (**E**) Schematic diagram of the transverse OLIF approach and operation.

*OLIF:* The patients were positioned in the lateral decubitus position on their right side after general anaesthesia was performed. A preoperative C-arm was used to locate the surgical segment and the centre of the target intervertebral disc (IVD). A 4–10 cm incision was made in the left anterior abdominal from approximately 3–4 cm anterior to the centre of the target. The proven online, internal oblique abs and rectus abdominis muscle was bluntly separated, and a retroperitoneal approach was adopted to expose the disc in the anterior psoas. A working channel was then installed. The disc was removed and cartilaginous endplate was scrapped, preserving the anterior and posterior annulus fibrosus. The contralateral annulus fibrosus was released and OLIF Cage was inserted (Medtronic, USA) (Figure 2). For those requiring posterior pedicle screw fixation, the prone position was taken after the anterior approach was performed the pedicle screws (Medtronic, USA) was inserted through a bilateral Wiltse approach with a bilateral small incision for single-segment or a posterior midline incision for multi-segments.

**Figure 3:**
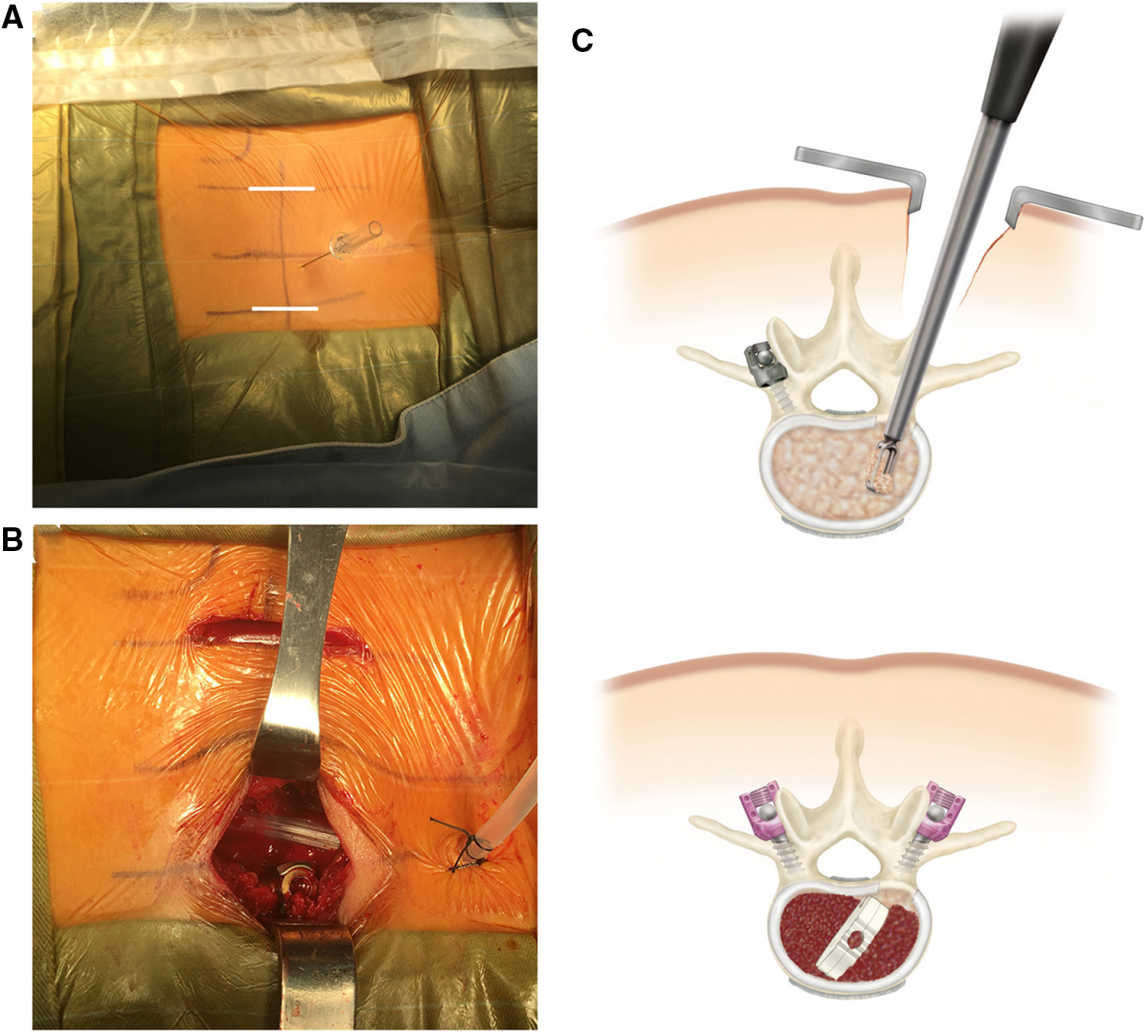
MIS-TLIF procedure (**A**) MIS-TLIF preoperative needle localization to determine the operative segment. The incision (white line) is located 2-3 cm away from the midline. (**B**) Bilateral small-incision MIS-TLIF, with the upper incision in the figure for pedicle screw placement *via* Wiltse approach and the lower incision for transforaminal decompression and intervertebral fusion. (**C**) Schematic diagram of the transverse MIS-TLIF approach and operation.

*MIS-TLIF*: After general anaesthesia, the patients were positioned in the prone position, a small bilateral incision for single-segment fusion or a posterior median incision for multi-segment fusion was made. The paravertebral muscles were routinely stripped along the spinous process on one side, and the Wiltse approach was accessed through the multifidus and longest muscle gap on the opposite side. Pedicle screws (Medtronic, Inc., USA) were implanted. The articular synovial joint on the striped side was removed. The disc and cartilaginous endplate were removed through the intervertebral foramen and the TLIF Cage was inserted (Medtronic, Inc., USA) (Figure 3).

### Postoperative management

Postoperative antibiotics were usually administered prophylactically for no more than 48 h. The anteroposterior and lateral x-rays of the lumbar spine were taken. On the first postoperative day, the patient could wear a lumbar brace and move out of bed under the guidance of the rehabilitation staff. The brace was worn for 3 months after surgery. The follow-up was performed routinely at 3, 6 and 12 months postoperatively.

### Evaluation indicators

**Figure 4:**
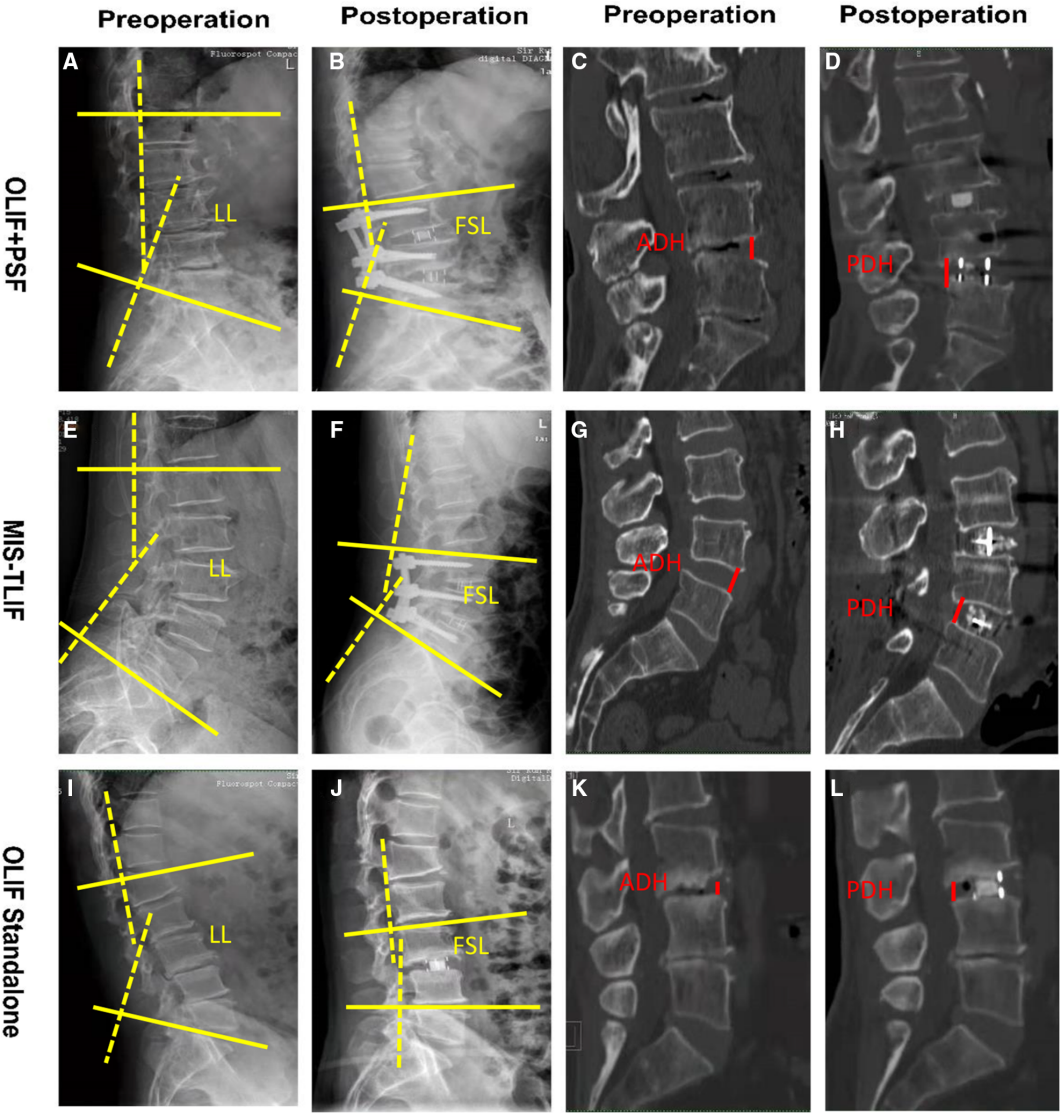
Schematic diagram of pre- and post-operative imaging parameter measurements. OLIF + PSF group: (**A,B**). Pre- and post-operative LL and FSL. (**C,D**) Pre- and post-operative ADH and PDH. MIS-TLIF group: (**E,F**) Pre- and post-operative LL and FSL of. (**G,H**) Pre- and post-operative ADH and PDH. OLIF Standalone group: (**I,J**) Pre- and post-operative LL and FSL. (**K,L**) Pre- and post-operative ADH and PDH.

The operation time, intraoperative bleeding and blood transfusion, complication, and fusion rate were recorded. The pain was evaluated by visual analogue score (VAS). The impact of lumbar pain on daily activities was evaluated by Oswestry dysfunction index (ODI) score before and after surgery. Fusion status was assessed by sagittally-reconstructed CT images about 1 year after the surgery. Lumbosacral lordosis (LL) was measured as the angle between the L1 superior endplate and S1 superior endplate on the lateral radiograph. Fused segmental anterior lordosis (FSL) was measured as the angle of intersection between the proximal fused supraspinal endplate and the distal fused supraspinal endplate on a lateral view. For example, if L3-S1 fusion was performed, FSL is the angle of intersection of the superior endplate of L3 and the superior endplate of S1 (Figure 4). ADH/PDH is the vertical distance of the measurement point of the anterior/posterior of the inferior endplate and the superior endplate of the disc of the fusion level (Figure 4). The measurement tool used for LL, FSL, ADH and PDH was Image J software (1.52u, National Institutes of Health, USA). All radiographic outcomes were evaluated by a blinded radiologist and a superior spine surgeon.

### Statistical analysis

All lumbosacral lordosis and disc height parameters were measured using Image J software. Statistical Packages of Social Sciences (SPSS) software (version 25.0) was used to analyze the collected data. The measurement data were expressed as $\bar{x}\pm s$, and the student t-test was used for comparison between OLIF + PSF, OLIF Standalone and MIS-TLIF groups. Count data were tested by the Chi-squared test or Fisher's exact test. *P *< 0.05 was considered a statistically significant difference.

## Results

### OLIF vs. MIS-TLIF

#### General conditions

All patients were followed up for 5 to 18 months, with a mean of 12.5 months. There were no statistically significant differences between the OLIF and MIS-TLIF groups in terms of gender, age, and the number of fused segments (all *P* > 0.05), while body mass index (BMI) was (22.9 ± 4.4) kg/m^2^ in the OLIF group, which was lower than that of (24.4 ± 5.0) kg/m^2^ in the MIS-TLIF group (*P* > 0.05). 20 cases (45.4%) in the OLIF group underwent posterior fixation, while all 39 cases (100%) in the TLIF group had posterior fixation (*χ*^2 ^= 41.013, *P *< 0.05) (Table 1).

**Table 1:** The comparison of general basic data of OLIF and MIS-TLIF.

Parameters	OLIF	MIS-TLIF	*P*
Sex, n, (M/F)	17/27	19/20	0.355
Age, years, mean + SD	66.7 ± 10.4	65.1 ± 11.3	0.505
BMI, kg/m^2^, mean + SD	22.9 ± 4.4	24.4 ± 5.0	0.155
Number of fusion Segments, n			0.445
Single segment	20	13	
Two segments	14	13	
Three segments	10	13	
Posterior PSF, *n*, (yes/no)	20/24	39/0	**0**.**000**
Operation time, min, mean + SD	189 ± 83	229 ± 80	**0**.**028**
Intraoperative bleeding, ml, mean ± SD	113 ± 138	421 ± 210	**0**.**000**
Blood transfusion, n, (yes/no)	3/41	10/29	**0**.**019**
**LL, deg, mean + SD**			
Preoperative	36.2 ± 14.2	41.0 ± 9.6	0.077
Postoperative	42.0 ± 12.4	45.0 ± 8.7	0.205
Correction	5.8 ± 9.8	4.0 ± 6.1	0.328
FSL Correction, deg, mean + SD	4.8 ± 7.2	4.9 ± 4.7	0.930
**ADH, mm, mean + SD**			
Preoperative	7.5 ± 1.26	7.6 ± 1.72	0.762
Postoperative	11.15 ± 3.68	9.03 ± 1.24	**0**.**001**
Increase	3.65 ± 2.42	1.43 ± 0.48	**0**.**000**
**PDH, mm, mean + SD**			
Preoperative	7.21 ± 1.29	6.97 ± 1.43	0.628
Postoperative	8.93 ± 2.20	7.96 ± 1.13	**0**.**015**
Increase	1.72 ± 0.91	0.99 ± 0.30	**0**.**000**
**VAS, mean + SD**			
Preoperative	6.3 ± 1.9	6.5 ± 1.7	0.628
Postoperative	1.7 ± 1.4	1.6 ± 1.3	0.952
Improvement	4.6 ± 1.9	4.9 ± 1.9	0.616
**ODI, mean + SD**			
Preoperative	58.5 ± 16.9	57.0 ± 19.1	0.705
Postoperative	19.4 ± 12.2	17.6 ± 13.1	0.504
Improvement	39.1 ± 16.4	39.4 ± 15.2	0.916
Fusion	42	34	0.728
Complication	5	3	0.717
Transient psoas weakness	3	0	
Thigh weakness or numbness	2	0	
Neurological injury	0	2	
Wound infection	0	1	

BMI, body mass index; LL, lumbar lordosis; FSL, fused segmental lordosis; ADH, Anterior disc height; PDH, Posterior disc height; VAS, visual analog score; ODI, Oswestry dysfunction score.

#### Operative time, intraoperative bleeding and blood transfusion

The operative time and intraoperative bleeding were significantly less in the OLIF group than in the MIS-TLIF group (both *P* < 0.001). 3 cases (6.8%) in the OLIF group and 10 cases (25.6%) in the MIS-TLIF group underwent blood transfusion, and the proportion of transfusion was significantly lower in the OLIF group than in the MIS-TLIF group ((*χ*2 = 5.545, *P* = 0.019) (Table 1).

After comparing the OLIF and MIS-TLIF groups according to the number of fused segments, it was found that the difference in operative time between the two groups for single-segment fusion was not statistically significant (*P* > 0.05), while the intraoperative bleeding was significantly less in the OLIF group than in the MIS-TLIF group (*P* < 0.05). In addition, the OLIF group had significantly less operative time and intraoperative bleeding for both double- and triple-segment fusion (all *P* < 0.05) (Table 2).

**Table 2:** The general comparison of OLIF and MIS-TLIF with different number of fused segments.

Parameters	OLIF	MIS-TLIF	*P*
Number	44	39	* *
**Single segment fusion**			
Operation time, min, mean ± SD	170 ± 75	167 ± 34	0.869
Intraoperative bleeding, ml, mean ± SD	87 ± 67	256 ± 161	**0**.**014**
Blood transfusion, n, (yes/no)	0/20	1/12	0.394
**LL, deg, mean + SD**			
Preoperative	41.6 ± 12.2	45.1 ± 11.8	0.417
Postoperative	45.8 ± 10.0	47.2 ± 10.9	0.715
Correction	4.2 ± 10.8	2.1 ± 4.1	0.495
FSL Correction, deg, mean + SD	4.1 ± 5.8	3.2 ± 4.3	0.647
ADH increase, mm, mean + SD	3.35 ± 2.13	1.23 ± 0.28	**0**.**000**
PDH increase, mm, mean + SD	1.62 ± 0.61	0.89 ± 0.21	**0**.**000**
**Two segments fusion**			
Operation time, min, mean ± SD	188 ± 84	210 ± 59	0.430
Intraoperative bleeding, ml, mean ± SD	98 ± 85	400 ± 100	**0**.**000**
Blood transfusion, n, (yes/no)	2/12	2/11	1.000
**LL, deg, mean + SD**			
Preoperative	36.6 ± 11.3	39.6 ± 6.6	0.402
Postoperative	43.5 ± 12.7	43.6 ± 7.7	0.981
Correction	6.9 ± 8.7	4.0 ± 6.0	0.326
FSL Correction, deg, mean + SD	5.6 ± 6.9	4.1 ± 4.7	0.529
ADH increase, mm, mean + SD	3.85 ± 2.62	1.43 ± 0.48	**0**.**000**
PDH increase, mm, mean + SD	2.32 ± 0.71	1.58 ± 0.15	**0**.**000**
**Three segments fusion**			
Operation time, min, mean ± SD	227 ± 89	309 ± 64	**0**.**018**
Intraoperative bleeding, ml, mean ± SD	239 ± 240	546 ± 207	**0**.**004**
Blood transfusion, n, (yes/no)	1/9	7/6	0.074
**LL, deg, mean + SD**			
Preoperative	25.0 ± 16.0	38.4 ± 9.2	**0**.**019**
Postoperative	32.3 ± 12.0	44.3 ± 7.3	**0**.**007**
Correction	7.2 ± 9.7	5.9 ± 7.5	0.706
FSL Correction, deg, mean + SD	5.0 ± 10.2	7.4 ± 4.4	0.468
ADH increase, mm, mean + SD	5.35 ± 2.32	2.41 ± 0.28	**0**.**000**
PDH increase, mm, mean + SD	3.72 ± 0.71	1.12 ± 0.41	**0**.**000**

#### Fusion and complication

As shown in Table 1, the OLIF group had higher overall fusion rates (42/44 OLIF vs. 34/39) compared with those in MIS-TLIF group, but the differences were not statistically significant (*P* = 0.728, Table 1). Both the OLIF group and MIS-TLIF group had lower overall complication rates (5/44 OLIF vs. 3/39 MIS-TLIF), the differences between the two groups were also not statistically significant (*P* = 0.717, Table 1). Among the patients in the OLIF group, two patients had intraoperative endplate injuries, two patients experienced transient psoas weakness and one patient experienced thigh numbness. There were three patients with complications (two with intraoperative endplate injury and one with neurological injury) in the MIS-TLIF group.

#### OLIF + PSF vs. MIS-TLIF and OLIF standalone

Comparing OLIF + PSF vs. MIS-TLIF, we found that the difference in operative time between the two groups was not statistically significant (*P* > 0.05), but intraoperative bleeding in the OLIF group was significantly less than that in the MIS-TLIF group (*P* < 0.05). While comparing OLIF combined with PSF vs. OLIF Standalone, there was a significant difference in operative time between the two groups (*P* < 0.05). However, the intraoperative bleeding of the two groups was not statistically different (Table 3).

**Table 3:** The general comparison of OLIF + PSF, OLIF standalone and MIS-TLIF.

Parameters	OLIF + PSF	MIS-TLIF	*P*	OLIF Standalone	*P*
Number, *n*	20	39		24	* *
Operation time, min, mean ± SD	243 ± 75	229 ± 80	0.513^a^	143 ± 59	**0**.**000^b^**
Intraoperative bleeding, ml, mean ± SD	128 ± 87	431 ± 210	**0**.**000^a^**	119 ± 168	0.845^b^
Blood transfusion, *n*, (yes/no)	2/18	10/29	0.192^a^	1/23	0.583^b^

^a^ *P* value of OLIF + PSF compared with MIS-TLIF.

^b^ *P* value of OLIF + PSF compared with OLIF Standalone.

#### Preoperative and postoperative VAS and ODI scores

In the OLIF group, VAS decreased from to 6.3 ± 1.9 preoperatively to 1.7 ± 1.4 postoperatively, and ODI decreased from 58.5 ± 16.9 preoperatively to 19.4 ± 12.2 postoperatively (both *P* < 0.05). In the MIS-TLIF group, VAS decreased from 6.5 ± 1.7 preoperatively to 1.6 ± 1.3 postoperatively, and ODI decreased from 57.0 ± 19.1 preoperatively to 17.6 ± 13.1 postoperatively (both *P* < 0.05). There were no statistically significant differences in VAS, ODI, postoperative VAS and ODI decline scores between the OLIF and MIS-TLIF groups before and after surgery (all *P* > 0.05) (Table 1).

#### Pre- and postoperative LL, FSL, ADH and PDH

No statistically significant difference was found between the pre- and postoperative LL and in MIS-TLIF group and the OLIF group (both *P* > 0.05). The LL correction was 5.8 ± 9.8 deg in the OLIF group and 4.0 ± 6.1 deg in the MIS-TLIF group (*P* > 0.05). The FSL correction was 4.8 ± 7.2 deg in the OLIF group and 4.9 ± 4.7 deg in the MIS-TLIF group (*P* > 0.05). The FSL correction was 4.1 ± 7.0 deg in the OLIF group and 5.2 ± 4.6 deg in the MIS-TLIF group (*P* > 0.05). There was no statistically significant difference in preoperative ADH and PDH between the OLIF group and MIS-TLIF group (*P* > 0.05). Nevertheless, the ADH and PDH increases were significantly higher in the OLIF group than in the MIS-TLIF group (both *P* < 0.001) (Table 1).

No statistically significant difference was found in LL and FSL correction between the two groups for single-segment fusion and double-segment fusion (all *P* > 0.05). For three- segment fusion, the preoperative and postoperative LL in the OLIF group was significantly smaller than that in the MIS- TLIF group (*t* = 1.831, 1.277, both *P* < 0.05), while the differences in the correction of LL and FSL were not statistically significant in both groups (*t* = 0.984, 0.088, both *P* > 0.05), while the differences in the correction of LL and FSL were not statistically significant in both groups (*t* = 0.186, 0.303, both *P* > 0.05). The OLIF group was significantly better than the MSI-TLIF group for the increase in ADH and PDH in single, dual or tri-segmental segments (all *P* < 0.05) (Table 2).

#### OLIF + PSF vs. OLIF standalone

**Figure 5:**
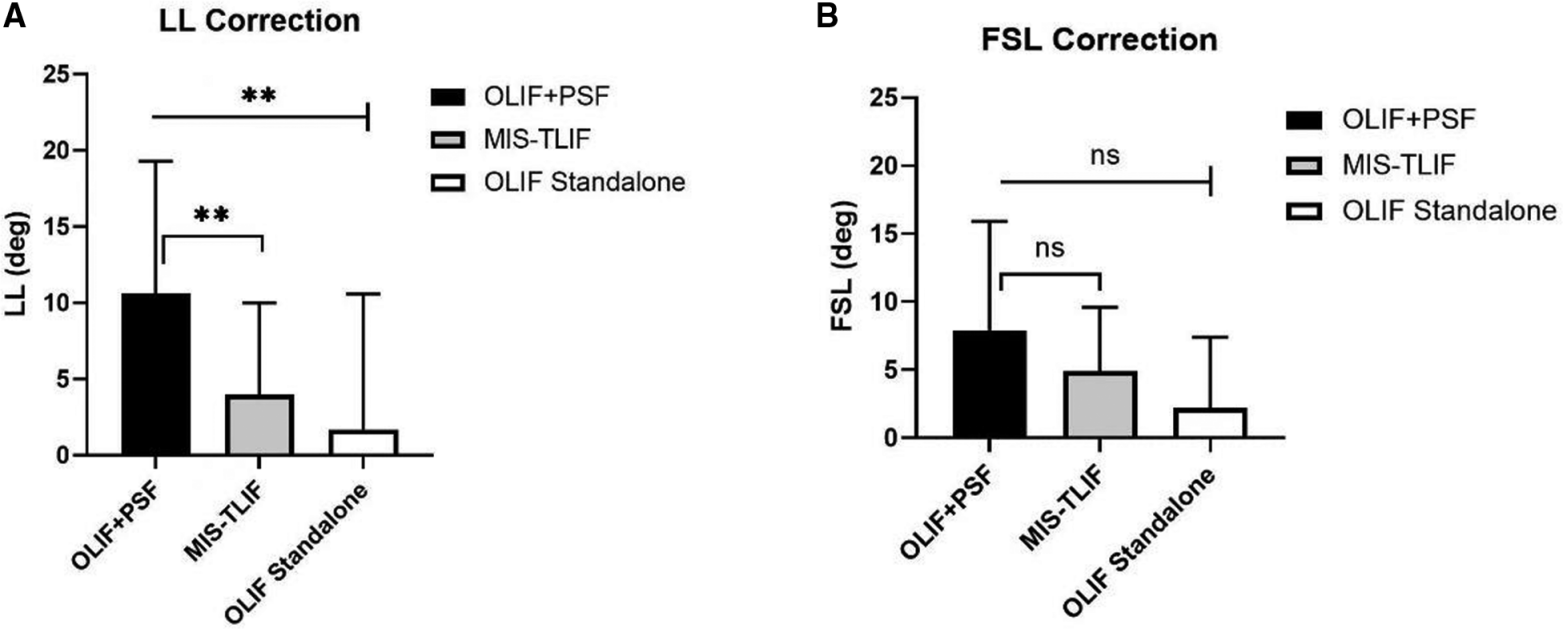
Post-operative correction of LL, FSL, in OLIF+PSF, OLIF Standalone and MIS-TLIF. A. LL Correction. B. FSL Correction.

The OLIF group was further divided into *OLIF + PSF* group and OLIF Standalone group based on whether posterior PSF was used, and it was found that the LL correction in OLIF + PSF group was significantly greater than that in the OLIF Standalone group (*P* < 0.05), while the FSL correction in OLIF + PSF group was also greater than the OLIF Standalone group, but the difference between the two groups was not statistically significant (*P* > 0.05). There were no statistical differences in ADH and PDH increase between OLIF + PSF and OLIF Standalone groups (*P* > 0.05) (Figure 5).

#### OLIF + PSF vs. MIS-TLIF

Comparing OLIF adding PSF with MIS-TLIF showed that the difference in preoperative LL was not statistically significant between the two groups (*P* > 0.05), but the correction of LL in the OLIF + PSF group was significantly greater than that in the MIS-TLIF group (*P* < 0.05). However, there was no statistically significant difference in the correction of FSL between the two groups (*P* > 0.05). The OLIF + PSF group showed a more significant increase in ADH and PDH compared to the MIS-TLIF group (both *P* < 0.001) (Figure 5).

## Discussion

For lumbar interbody fusion (LIF), the anterior/lateral approach has a natural access advantage vs. the posterior approach (5). Differs from the posterior approach, which requires muscle stripping and access to the spinal canal, the anterior/lateral approach allows direct access to the target disc *via* the posterior peritoneal space, thus greatly reducing the damage caused by the operation and decreasing the risk of bleeding (4, 7). At the same time, the anterior/lateral approach can reach most of the lumbar intervertebral discs *via* the same anatomic space, which is particularly suitable for multi-segment fusion. As reflected in the results of this study, the OLIF operative time was significantly shorter than MIS-TLIF, which was particularly prominent in multi-segmental fusion. Regarding intraoperative bleeding, OLIF was superior to MIS-TLIF in both single and multi-segment fusions. Similar results have been reported by other authors (8–11). In terms of clinical outcomes, both OLIF and MIS-TLIF significantly reduced pain and improved dysfunctional conditions, but there were no significant differences between the two groups, reflecting the satisfactory short-term clinical efficacy of OLIF as a new technique. For radiological correction, both OLIF and MIS-TLIF can restore lumbosacral lordosis and disc height partly. Nevertheless, OLIF is more effective than MIS-TLIF in restoring anterior and posterior intervertebral disc heights (ADH and PDH). Whilst, OLIF combined with PSF can acquire a better correction effect for lumbosacral lordosis than MIS-TLIF. The OLIF group had higher overall fusion rates compared to those in the MIS-TLIF group. This is because the OLIF procedure uses a larger cage with a larger contact area with the endplate. The OLIF procedure has a slightly steeper learning curve and a theoretically higher complication rate than MIS-TLIF. Although the complication rate was somewhat higher in the OLIF group, in our study, the surgeon was proficient in both procedures. Therefore, both the OLIF group and MIS-TLIF group had low overall complication rates.

Despite some controversy, many studies have suggested that OLIF obtains similar clinical effects to MIS-TLIF (12). Different from MIS-TLIF, OLIF achieves neural decompression indirectly by implanting an enlarged cage from a window between the lateral border of major vessels and the psoas muscle (13). As OLIF does not interfere with the spinal canal and nerve roots, the procedure may further alleviate symptoms of nerve pain and low back pain by enlarging the dimension of the spinal canal and intervertebral foramen (14). On the contrary, the MIS-TLIF opens the spinal canal and intervertebral foramen directly for decompression, which is also effective in relieving pain caused by nerve compression (15). The present study showed that although there was no statistical difference between OLIF and MIS-TLIF, MIS-TLIF still showed an advantage in reducing neuropathic pain, which is generally consistent with the results of previous studies. Some surgeons have suggested that this advantage may be attributed to the direct and effective decompression of the nerve by MIS-TLIF (16). Regarding functional recovery, improvement of the ODI scores was also similar between OLIF and MIS-TLIF. Similar efficacy was confirmed through different evaluation indicators for example Japanese Orthopedic Association Back Pain Evaluation Questionnaire (JOABPEQ), Physical Function and Quality of Life (QOL) et al. by other researchers (17, 18).

Restoration of sagittal balance and correction of lumbosacral lordosis is crucial to the outcome of lumbar interbody fusion (19), and the most important factor affecting the outcome of lumbosacral lordosis correction is the restoration of disc height (3, 20). The anterior approach allows the implantation of a larger cage than the posterior approach, thus theoretically facilitating the recovery of the lordosis angle more (5). The literature reports that ALIF is superior to LLIF and TLIF in the correction of lumbar lordosis (21)), mainly due to the ability of ALIF to remove the anterior longitudinal ligament and the anterior fibrous annulus, thus providing relatively complete release of the anterior disc structures. However, due to the anatomical characteristics of the lumbar spine adjacent to major blood vessels, ALIF is only relatively applicable to the L4/5 and L5-S1 segments, and it is very difficult to expose in the L3/4 and higher segments, which can easily cause damage to macrovascular (22). Compared to ALIF, OLIF is more widely applicable to all discs between L1 and L5 (5). In addition, compared to XLIF/DLIF, OLIF has a much lower risk of injury to the psoas muscle muscles and lumbar plexus (6). Therefore, OLIF is our choice for performing anterior intervertebral fusion, even for the L5 to S1 segment (23).

Theoretically, OLIF is more effective than MIS-TLIF in restoring disc height. Several retrospectives and prospective comparative studies have shown that OLIF provides better restoration of disc height compared to MIS-TLIF (12, 17). Our results likewise showed that the increase in ADH and PDH in OLIF overall and OLIF with additional PSF group were greater than MIS-TLIF group, whereas there was no significant difference in ADH and PDH between OLIF with additional PSF and OLIF Standalone.

Furthermore, some studies have shown that TLIF does not restore anterior lumbar lordosis (2, 9, 11), but others have shown that the correction of lordosis by TLIF depends on the degree of posterior column shortening (10, 13). The results of the present study show that MIS-TLIF has some degree of correction effect for lumbosacral lordosis, which may be related to the fact that we performed MIS-TLIF with as large a cage as possible and with a considerable degree of pressurized shortening of the posterior column structure. Compared with TLIF, we expected a better performance of OLIF in terms of lumbosacral lordosis correction, because previous literature generally reported that LLIF was effective in restoring lumbar lordosis and the lordosis angle of the fused segment (9, 10, 14, 15). However, the results of the present study showed that OLIF did not have a significant advantage over MIS-TLIF in restoring global lumbosacral lordosis and fused segmental lordosis angles. We believe this may be related to the fact that posterior pedicle fixation was not used in about 50% of the OLIF cases in this study. Yson et al. (16) reported that posterior pedicle fixation could add about 1 deg of lordosis correction based on placement of the lateral interbody cage. The present study similarly found that OLIF combined with PSF restored more lordosis angle than OLIF Standalone. Thus, the placement of a large interbody cage anteriorly to distract the intervertebral space needs to be supplemented with PSF compression to achieve greater lumbosacral lordosis correction with OLIF.

The reasons for the results described in the previous section are that larger cages were used in OLIF (width up to 55 mm and height up to 14 mm), while MIS-TLIF cages are relatively small (width of 25–30 mm and height up to 10–12 mm)(Lin et al., 2018). During the OLIF procedure, the wide cage is placed on the solid epiphyseal ring around the vertebral body, rather than on the relatively vulnerable area of the bone cortical in the central concave of the endplate, so the distraction of intervertebral space is more effective (24). In addition, another important fact is that 6 deg or 12 deg cage is used in OLIF, but the 0 deg cage is used in MIS-TLIF (16, 25).

Because posterior pedicle screw fixation has a specific lordosis correction effect, comparing OLIF containing mostly OLIF Standalone with MIS-TLIF may underestimate the lordosis correction effect of OLIF produced by the cage. We then further compared the ability of the OLIF adding with PSF with the MIS-TLIF group in terms of lordosis correction and disc height increase. We found that greater lumbosacral lordosis correction was obtained with OLIF than MIS-TLIF with the same use of posterior pedicle screw fixation. However, the increase in intervertebral height (ADH and PDH) was similar in both OLIFs with PSF and OLIF alone. Consequently, OLIF has some inherent advantages over MIS-TLIF in terms of restoring the lumbosacral lordosis and disc height.

There are limitations to this study. Firstly, the selection of the procedure is subjective and cannot be completely standardized, and selection bias cannot be avoided. Secondly, this is a lack of long-term follow-up data. Thirdly, the procedure is not done by the same surgeon, and there is a learning curve early in the development of OLIF, which may cause differences in the outcome of the same procedure. Finally, the disease spectrum is wide, the dispersion of baseline imaging data indices is large, and the indications for surgery are not the same between the two groups.

In overview, both OLIF and TLIF can restore the lumbosacral lordosis angle to some extent, but OLIF has a significant advantage regarding operative time and intraoperative bleeding. With the addition of PSF, OLIF provided better results for lumbosacral lordosis correction than MIS-TLIF, and the higher and wider cage of OLIF may account for this difference. This implies that OLIF may be more suitable than MIS-TLIF for the treatment of degenerative scoliosis. In addition, the addition of posterior pedicle screw fixation has greatly improved the ability of OLIF to restore the lordosis angle, while the ability of OLIF Standalone to correct the lordosis is more limited. Therefore, the addition of posterior screw fixation or even shortening of the posterior column remains necessary in cases of degenerative scoliosis in which correction of the sagittal plane deformity is the primary treatment goal.

## Data Availability

The original contributions presented in the study are included in the article/Supplementary Material, further inquiries can be directed to the corresponding author/s.
