# Corrigendum: Can oblique lateral interbody fusion (OLIF) create more lumbosacral lordosis in lumbar spine surgery than minimally invasive transforaminal interbody fusion (MIS-TLIF)?

**DOI:** 10.3389/fsurg.2023.1171387

**Published:** 2023-04-28

**Authors:** Jie Li, Yilei Chen, Hao Wu, Kaifeng Gan, Dikai Bei, Tengdi Fan, Jian Chen, Binhui Chen

**Affiliations:** ^1^ Department of Spine Surgery, Ningbo Medical Centre Li Huili Hospital, Ningbo, China; ^2^ Department of Orthopaedic Surgery, Sir Run Run Shaw Hospital, Hangzhou, China; ^3^ Department of Orthopaedics and Traumatology, Faculty of Medicine, The Chinese University of Hong Kong, Shatin, China

**Keywords:** OLIF, posterior pedicle screw fixation, OLIF standalone, lumbar degenerative disease, lumbosacral lordosis

A corrigendum on Can oblique lateral interbody fusion (OLIF) create more lumbosacral lordosis in lumbar spine surgery than minimally invasive transforaminal interbody fusion (MIS-TLIF)? By Li J, Chen Y, Wu H, Gan K, Bei D, Fan T, Chen J and Chen B. (2023) Front. Surg. 10:1063354. doi: 10.3389/fsurg.2022.1063354

**Error in Figure/Table**

In the published article, there was data errors in [TABLE 1 The comparison of general basic data of OLIF and MIS-TLIF] as published. [The data in column “age”, “BMI”, “Operation time”, “Intraoperative bleeding”, “LL, deg,”, “FSL Correction”, “VAS” and “ODI” were incorrectly calculated.]. The corrected [TABLE 1 The comparison of general basic data of OLIF and MIS-TLIF] appear below.

**Table 1:** The comparison of general basic data of OLIF and MIS-TLIF.

Parameters	OLIF	MIS-TLIF	*P*
Sex, n, (M/F)	17/27	19/20	0.355
Age, years, mean + SD	66.7 ± 10.4	65.1 ± 11.3	0.505
BMI, kg/m^2^, mean + SD	22.9 ± 4.4	24.4 ± 5.0	0.155
Number of fusion Segments, n			0.445
Single segment	20	13	
Two segments	14	13	
Three segments	10	13	
Posterior PSF, *n*, (yes/no)	20/24	39/0	**0**.**000**
Operation time, min, mean + SD	189 ± 83	229 ± 80	**0**.**028**
Intraoperative bleeding, ml, mean ± SD	113 ± 138	421 ± 210	**0**.**000**
Blood transfusion, n, (yes/no)	3/41	10/29	**0**.**019**
**LL, deg, mean + SD**			
Preoperative	36.2 ± 14.2	41.0 ± 9.6	0.077
Postoperative	42.0 ± 12.4	45.0 ± 8.7	0.205
Correction	5.8 ± 9.8	4.0 ± 6.1	0.328
FSL Correction, deg, mean + SD	4.8 ± 7.2	4.9 ± 4.7	0.930
**ADH, mm, mean + SD**			
Preoperative	7.5 ± 1.26	7.6 ± 1.72	0.762
Postoperative	11.15 ± 3.68	9.03 ± 1.24	**0**.**001**
Increase	3.65 ± 2.42	1.43 ± 0.48	**0**.**000**
**PDH, mm, mean + SD**			
Preoperative	7.21 ± 1.29	6.97 ± 1.43	0.628
Postoperative	8.93 ± 2.20	7.96 ± 1.13	**0**.**015**
Increase	1.72 ± 0.91	0.99 ± 0.30	**0**.**000**
**VAS, mean + SD**			
Preoperative	6.3 ± 1.9	6.5 ± 1.7	0.628
Postoperative	1.7 ± 1.4	1.6 ± 1.3	0.952
Improvement	4.6 ± 1.9	4.9 ± 1.9	0.616
**ODI, mean + SD**			
Preoperative	58.5 ± 16.9	57.0 ± 19.1	0.705
Postoperative	19.4 ± 12.2	17.6 ± 13.1	0.504
Improvement	39.1 ± 16.4	39.4 ± 15.2	0.916
Fusion	42	34	0.728
Complication	5	3	0.717
Transient psoas weakness	3	0	
Thigh weakness or numbness	2	0	
Neurological injury	0	2	
Wound infection	0	1	

BMI, body mass index; LL, lumbar lordosis; FSL, fused segmental lordosis; ADH, Anterior disc height; PDH, Posterior disc height; VAS, visual analog score; ODI, Oswestry dysfunction score.

In the published article, there was data errors in [TABLE 2 The general comparison of OLIF and MIS-TLIF with different number of fused segments] as published. [The data in column “Operation time”, “Intraoperative bleeding”, “LL, deg,”, “FSL Correction” were incorrectly calculated.]**.** The corrected [TABLE 2 The general comparison of OLIF and MIS-TLIF with different number of fused segments] appear below.

**Table 2:** The general comparison of OLIF and MIS-TLIF with different number of fused segments.

Parameters	OLIF	MIS-TLIF	*P*
Number	44	39	* *
**Single segment fusion**			
Operation time, min, mean ± SD	170 ± 75	167 ± 34	0.869
Intraoperative bleeding, ml, mean ± SD	87 ± 67	256 ± 161	**0**.**014**
Blood transfusion, n, (yes/no)	0/20	1/12	0.394
**LL, deg, mean + SD**			
Preoperative	41.6 ± 12.2	45.1 ± 11.8	0.417
Postoperative	45.8 ± 10.0	47.2 ± 10.9	0.715
Correction	4.2 ± 10.8	2.1 ± 4.1	0.495
FSL Correction, deg, mean + SD	4.1 ± 5.8	3.2 ± 4.3	0.647
ADH increase, mm, mean + SD	3.35 ± 2.13	1.23 ± 0.28	**0**.**000**
PDH increase, mm, mean + SD	1.62 ± 0.61	0.89 ± 0.21	**0**.**000**
**Two segments fusion**			
Operation time, min, mean ± SD	188 ± 84	210 ± 59	0.430
Intraoperative bleeding, ml, mean ± SD	98 ± 85	400 ± 100	**0**.**000**
Blood transfusion, n, (yes/no)	2/12	2/11	1.000
**LL, deg, mean + SD**			
Preoperative	36.6 ± 11.3	39.6 ± 6.6	0.402
Postoperative	43.5 ± 12.7	43.6 ± 7.7	0.981
Correction	6.9 ± 8.7	4.0 ± 6.0	0.326
FSL Correction, deg, mean + SD	5.6 ± 6.9	4.1 ± 4.7	0.529
ADH increase, mm, mean + SD	3.85 ± 2.62	1.43 ± 0.48	**0**.**000**
PDH increase, mm, mean + SD	2.32 ± 0.71	1.58 ± 0.15	**0**.**000**
**Three segments fusion**			
Operation time, min, mean ± SD	227 ± 89	309 ± 64	**0**.**018**
Intraoperative bleeding, ml, mean ± SD	239 ± 240	546 ± 207	**0**.**004**
Blood transfusion, n, (yes/no)	1/9	7/6	0.074
**LL, deg, mean + SD**			
Preoperative	25.0 ± 16.0	38.4 ± 9.2	**0**.**019**
Postoperative	32.3 ± 12.0	44.3 ± 7.3	**0**.**007**
Correction	7.2 ± 9.7	5.9 ± 7.5	0.706
FSL Correction, deg, mean + SD	5.0 ± 10.2	7.4 ± 4.4	0.468
ADH increase, mm, mean + SD	5.35 ± 2.32	2.41 ± 0.28	**0**.**000**
PDH increase, mm, mean + SD	3.72 ± 0.71	1.12 ± 0.41	**0**.**000**

In the published article, there was data errors in [Table 3 The General comparison of OLIF + PSF, OLIF Standalone and MIS-TLIF] as published. [The data in column “Operation time”, “Intraoperative bleeding” were incorrectly calculated.]. The corrected [Table 3 The General comparison of OLIF + PSF, OLIF Standalone and MIS-TLIF] appear below.

**Table 3:** The general comparison of OLIF + PSF, OLIF standalone and MIS-TLIF.

Parameters	OLIF + PSF	MIS-TLIF	*P*	OLIF Standalone	*P*
Number, *n*	20	39		24	* *
Operation time, min, mean ± SD	243 ± 75	229 ± 80	0.513^a^	143 ± 59	**0**.**000^b^**
Intraoperative bleeding, ml, mean ± SD	128 ± 87	431 ± 210	**0**.**000^a^**	119 ± 168	0.845^b^
Blood transfusion, *n*, (yes/no)	2/18	10/29	0.192^a^	1/23	0.583^b^

In the published article, there was data errors in [Figure 5. Post-operative correction of LL, FSL, in OLIF + PSF, OLIF Standalone and MIS-TLIF. A. LL Correction. B. FSL Correction] as published. [The column length was changed because of incorrectly calculated data]**.** The corrected [Figure 5. Post-operative correction of LL, FSL, in OLIF + PSF, OLIF Standalone and MIS-TLIF. A. LL Correction. B. FSL Correction] appear below.


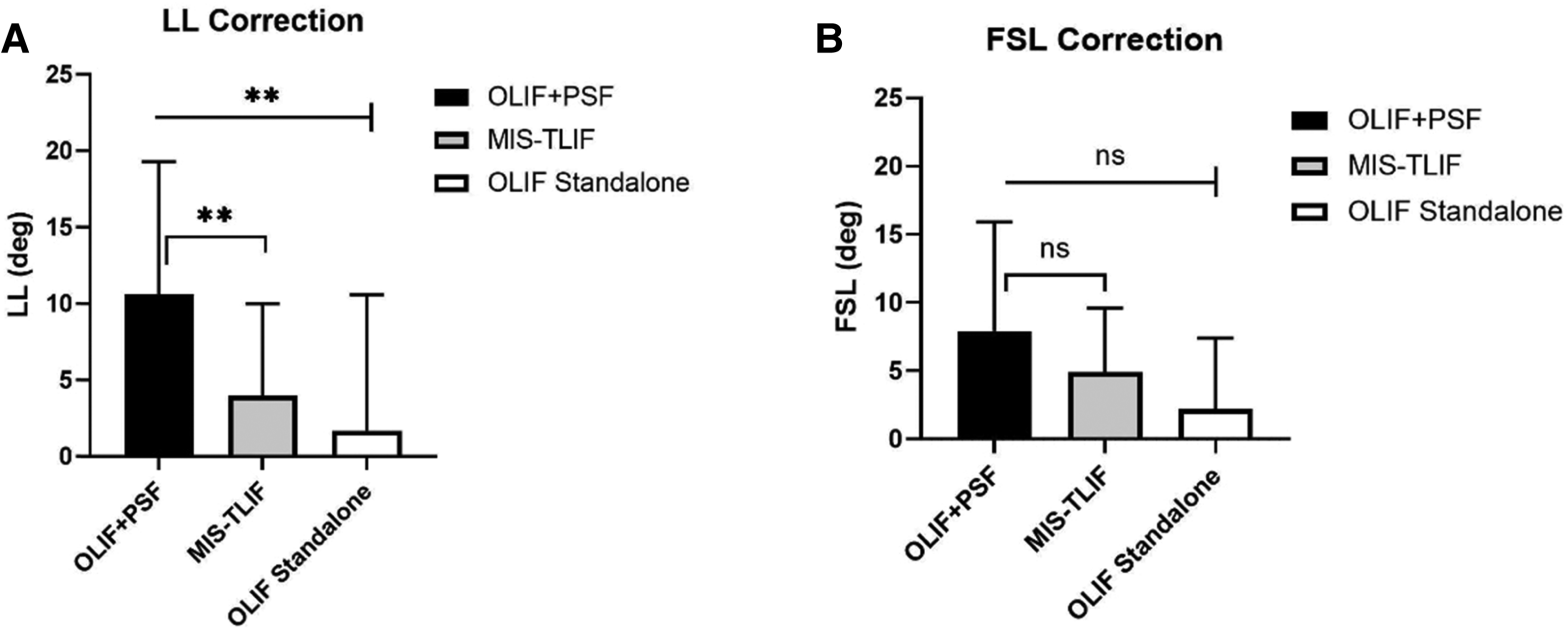


The authors apologize for this error and state that this does not change the scientific conclusions of the article in any way. The original article has been updated.

**Text Correction**

In the published article, there was an error. [Some data was incorrectly calculated].

A correction has been made to **[Abstract]**, *[Method***]**, [Paragraph 1]. This sentence previously stated:

“[…mean age 65.8 years…]”

The corrected sentence appears below:

“[…mean age 66.0 ± 10.8 years…]”

A correction has been made to **[Abstract]**, [*Results***]**, [Paragraph 1]. This sentence previously stated:

“[The average operation time and intraoperative bleeding were significantly less in the OLIF group than in the MIS-TLIF group (163 ± 68 vs. 233 ± 79 min, 116 ± 148 vs. 434 ± 201 ml, *P* < 0.001)……. The correction of LL was significantly more in the OLIF + PSF group than in the MIS-TLIF group (9.9 ± 11.1 vs. 4.2 ± 6.1deg, *P* = 0.034).]”

The corrected sentence appears below:

“[The average operation time and intraoperative bleeding were significantly less in the OLIF group than in the MIS-TLIF group (189 ± 83 vs. 229 ± 80 min, 113 ± 138 vs. 421 ± 210 ml). ……. The correction of LL was significantly more in the OLIF + PSF group than in the MIS-TLIF group (10.6 ± 8.7 vs. 4.0 ± 6.1 deg, *P* = 0.005).]”

A correction has been made to **[Results]**, *[Name of Sub-section if there is one***]**, [Paragraph Number]. This sentence previously stated:

“[…while body mass index (BMI) was (23.6 ± 2.8) kg/m^2^ in the OLIF group, which was lower than that of (25.0 ± 3.1) kg/m^2^ in the MIS-TLIF group (*P* < 0.05).]”

The corrected sentence appears below:

“[…while body mass index (BMI) was (22.9 ± 4.4) kg/m^2^ in the OLIF group, which was lower than that of (24.4 ± 5.0) kg/m^2^ in the MIS-TLIF group (*P* > 0.05)]”

A correction has been made to **[Results]**, *[Operative time, intraoperative bleeding and blood transfusion***]**, [Paragraph 1]. This sentence previously stated:

“[…((*χ*^2^ = 5.545, *P* = 0.019)]”

The corrected sentence appears below:

“[…((*χ*^2^ = 5.545, *P* = 0.019)]”

A correction has been made to **[Results]**, *[Preoperative and postoperative VAS and ODI scores***]**, [Paragraph 1]. This sentence previously stated:

“[In the OLIF group, VAS decreased from to 6.2 ± 2.0 preoperatively to 2.0 ± 1.3 postoperatively, and ODI decreased from 50 ± 18 preoperatively to 15 ± 10 postoperatively (both *P* < 0.05). In the MIS-TLIF group, VAS decreased from 7.0 ± 1.1 preoperatively to 1.6 ± 1.3 postoperatively, and ODI decreased from 56 ± 16 preoperatively to 17 ± 15 postoperatively (both *P* < 0.05).]”

The corrected sentence appears below:

“[In the OLIF group, VAS decreased from to 6.3 ± 1.9 preoperatively to 1.7 ± 1.4 postoperatively, and ODI decreased from 58.5 ± 16.9 preoperatively to 19.4 ± 12.2 postoperatively (both *P* < 0.05). In the MIS-TLIF group, VAS decreased from 6.5 ± 1.7 preoperatively to 1.6 ± 1.3 postoperatively, and ODI decreased from 57.0 ± 19.1 preoperatively to 17.6 ± 13.1 postoperatively (both *P* < 0.05).]”

A correction has been made to **[Results]**, *[Pre- and postoperative LL, FSL, ADH and PDH e***]**, [Paragraph 1]. This sentence previously stated:

“[The LL correction was 4.0 ± 10.0 deg in the OLIF group and 4.2 ± 6.1 deg in the MIS-TLIF group (*P* > 0.05). The FSL correction was 4.1 ± 7.0 deg in the OLIF group and 5.2 ± 4.6 deg in the MIS-TLIF group (*P* > 0.05).]”

The corrected sentence appears below:

“[The LL correction was 5.8 ± 9.8 deg in the OLIF group and 4.0 ± 6.1 deg in the MIS-TLIF group (*P* > 0.05). The FSL correction was 4.8 ± 7.2 deg in the OLIF group and 4.9 ± 4.7 deg in the MIS-TLIF group (*P* > 0.05).]”

A correction has been made to **[Results]**, *[Pre- and postoperative LL, FSL, ADH and PDH***]**, [Paragraph 2]. This sentence previously stated:

“[For three-segment fusion, the preoperative and postoperative LL in the OLIF group was significantly smaller than that in the MISTLIF group (*t* = 2.190, 2.661, both *P* < 0.05), while the differences in the correction of LL and FSL were not statistically significant in both groups (*t* = 0.186, 0.303, both *P* > 0.05).]”

The corrected sentence appears below:

“[For three- segment fusion, the preoperative and postoperative LL in the OLIF group was significantly smaller than that in the MIS- TLIF group (*t* = 1.831, 1.277, both *P* < 0.05), while the differences in the correction of LL and FSL were not statistically significant in both groups (*t* = 0.984, 0.088, both *P* > 0.05).]”

The authors apologize for this error and state that this does not change the scientific conclusions of the article in any way. The original article has been updated.

